# Transactions Texas State Medical Association

**Published:** 1896-11

**Authors:** 


Transactions Texas State Medical Association for 1896, 28th annual session; Fort Worth meeting. The Journal has received a copy of the Transactions just issued from the press of Von Boeckmann, of Austin. The contract for the work, it will be remembered, was awarded to Von Boeckmann on competitive bid, and though it is one of the very best jobs that has ever been turned out for the Texas Medical Association, it cost far less than any that has been issued within recent years.

The book is printed in clear new Roman type on a superior quality of book paper, and is a clean, neat, compact and well executed job, mechanically. The Association surely will be satisfied with it especially considering the great saving in the cost over volumes printed in Galveston. It is neatly bound in paper covers, and contains 468 pages, cost about 79 cents per volume. Discussions and resolutions and certain other parts are printed in brevier. The frontispiece is an engraving of the President, Dr. J. C. Loggins, of Ennis.

Of the character of the papers, we cannot speak at present, not having had opportunity yet to examine them, but at the close of the meeting it was generally admitted that the papers were of a high order of excellence.

